# Effects of Single-Task, Dual-Task and Analogy Training during Gait Rehabilitation of Older Adults at Risk of Falling: A Randomized Controlled Trial

**DOI:** 10.3390/ijerph20010315

**Published:** 2022-12-25

**Authors:** Toby C. T. Mak, Catherine M. Capio, Thomson W. L. Wong

**Affiliations:** 1 Department of Rehabilitation Sciences, The Hong Kong Polytechnic University, Hong Kong SAR, China; 2 Centre for Educational and Developmental Sciences, The Education University of Hong Kong, Hong Kong SAR, China; 3 Department of Health Science, Ateneo de Manila University, Quezon City 1108, Philippines

**Keywords:** single-task, dual-task, analogy, physical wellbeing, older adults

## Abstract

It has been suggested that implicit motor learning via dual-task or analogy training during gait rehabilitation may yield better outcomes in older adults by reducing the propensity for the conscious processing of movements (movement-specific reinvestment). The current study investigated the immediate effects of single-task, dual-task, and analogy training on reinvestment propensity and fall-related rehabilitation outcomes among older adults at risk of falling. Seventy-one older adults were randomly allocated to the single-task (ST), dual-task (DT), or analogy (AG) training conditions and received 12 training sessions. We assessed the reinvestment propensity, functional gait and balance, functional mobility, balance ability, single-task and dual-task walking abilities, and fear of falling at baseline (before training) and immediately after training. Our findings revealed a lack of training effect on reinvestment propensity for all groups. However, all groups displayed significant improvements in functional gait and balance (*p* < 0.001), functional mobility (*p* = 0.02), and balance ability (*p* = 0.01) after training. AG appeared to be superior to DT and ST, as it was the only condition that resulted in significant improvements in both single-task and dual-task walking abilities (*p* < 0.001). Implementing movement analogies could be a feasible and useful gait rehabilitation strategy for fall prevention and wellbeing promotion among older adults.

## 1. Introduction

Falls constitute a concern that can undermine the wellbeing of the older population. Falls can lead to serious physical injuries, such as hip fractures, hospitalization, and death [1]as well as wellbeing issues, including heightened fear of falling and reduced quality of life [2,3]. Problems with balance and gait are considered to be some of the most significant risk factors for falls [4]. This might be attributed to the fact that the majority of falls occur during walking [5]. Stable posture and gait are necessary for older adults to perform activities of daily living independently. Thus, training approaches that can potentially improve the balance and walking ability of older adults with a high risk of falling contribute to establishing effective fall prevention programs.

Healthy older adults generally walk with automaticity [6]. However, when under stress to avoid falling or encountering movement difficulties, older individuals—especially those with a high risk of falling—will likely adopt a more conscious manner of walking. This is typically characterized by the use of working memory resources to manipulate explicit, declarative knowledge to control movement mechanics [7,8]. The shift from relative automaticity to conscious monitoring and controlling the mechanics of movement has been referred to as ‘movement-specific reinvestment’ [7]. Previous research has indicated that older individuals with a history of falls have a higher propensity for the conscious processing of movement (i.e., movement-specific reinvestment) than age-matched non-fallers [9]. Such an association between movement-specific reinvestment propensity and the risk of falling might be explained, at least in part, by evidence that the conscious processing of movement could lead to compromised walking performance in older adults (possibly by interfering with automatic motor control) [10,11].

Therefore, research has emerged to explore methods that aim to reduce the propensity for movement-specific reinvestment to generate better outcomes (e.g., functional balance, walking ability) in the context of gait rehabilitation. Potential training approaches to mitigate movement-specific reinvestment propensity have been developed from the concept of implicit motor learning. In these approaches, the accrual of explicit rules and the cognitive involvement of working memory are deliberately suppressed [12]. Implicit learning refers to a process of acquiring information without the awareness of both the process and the outcome of knowledge acquisition, while explicit learning requires the awareness and acquisition of explicit rules [13]. Motor skills can be acquired in an implicit or explicit manner [14,15].

One type of implicit motor learning is via dual-task training, wherein a concurrent cognitive secondary task is performed during a primary motor task [14,16]. Dual-task training has been suggested to reduce the movement-specific reinvestment propensity by distracting or preventing individuals from reinvesting in the movement execution of the primary motor task, thus allowing automatic motor processes to dominate [14,16]. A systematic review summarized evidence showing advantages in cognitive and motor performance when dual-task training (with concurrent cognitive secondary tasks) for balancing or walking was performed over single-task training in the older population [17]. Another type of implicit motor learning is via analogy training, where a simple biomechanical metaphor that compares the movements with a similar well-known concept is used [18]. Researchers have argued that movement analogies minimize the accumulation of explicit knowledge of the underlying rules governing movement mechanics, thereby preventing an overload of working memory resources during motor learning [18]. Analogy training might be particularly useful for older adults, as the capacity for cognitive resources declines with age [19,20]. Movement analogies have indeed been shown to be beneficial for skill learning (e.g., table tennis) in the older adult population [21].

However, to the best of our knowledge, research has yet to investigate (and compare) the training effects of different implicit motor learning methodologies on movement-specific reinvestment propensity and fall-related rehabilitation outcomes among community-dwelling older adults. Such knowledge serves as critical evidence for physiotherapists in geriatric rehabilitation who aim to restore, maintain, or improve daily locomotion and independent function in older adults [22]. Exploring the potential impact of dual-task or analogy training on rehabilitation outcomes could provide practical insights for the development of future therapeutic interventions for fall prevention programs. For instance, physiotherapists might consider modifying instructions for older adults during gait re-education by utilizing analogies or concurrent cognitive secondary tasks.

The current study aimed to examine the immediate effects of dual-task and analogy training on movement-specific reinvestment propensity and fall-related outcomes in the context of gait rehabilitation among older adults at risk of falling. We hypothesized that at-risk older adults who complete dual-task or analogy training will display a greater reduction in movement-specific reinvestment propensity and greater improvements in fall-related rehabilitation outcomes than those who complete single-task training (active control group). The outcomes include functional gait and balance, balance ability, functional mobility, single-task and dual-task walking abilities, and reduced fear of falling.

## 2. Materials and Methods

### 2.1. Design

This study consists of a three-arm, parallel group, randomized controlled trial (RCT). Participants were randomly allocated into a single-task (ST) (active control), a dual-task (DT), or an analogy (AG) walking training group. The study protocol was published [23] and preregistered at ClinicalTrials.gov (NCT03811782).

### 2.2. Participants

Participants were recruited from nine elderly community centers in Hong Kong. Community-dwelling older adults were recruited by convenience sampling based on the following inclusion criteria: (1) age 65 or above; (2) able to walk independently indoors for a minimum of 10 m; (3) a total score of 24 or higher out of 30 in the Chinese version of the Mini-Mental State Examination (MMSE-C) [24,25]; (4) no history of untreated major neurological, vestibular, or musculoskeletal disorder (e.g., Parkinson’s disease or stroke); and (5) a total score of less than 24 out of 28 in the Tinetti Performance Oriented Mobility Assessment (POMA) [26]. The study was approved by the institutions’ human research ethics committee (Institutional Review Board of the University of Hong Kong/Hospital Authority Hong Kong West Cluster (IRB no.: UW17-049) and the Human Research Ethics Committee of the Education University of Hong Kong (E2019-2020-0040)), and informed consent was acquired from all participants before the start of any procedure.

### 2.3. Intervention

The training groups were differentiated by the walking instructions that were provided. An expert group of physiotherapists that specialized in geriatric rehabilitation designed the instructions. To ensure that participants were able to learn and understand the instructions comprehensively, all participants took part in their own group’s pre-training session (i.e., prior to the commencement of 12 training sessions), where they were clearly briefed about the specific walking instruction assigned to them.

In the ST, participants were instructed using typical therapeutic walking instructions (e.g., *“please walk with bigger steps and even weight-bearing on your two feet”*). The instructions, as a common practice, were dependent on participants’ walking ability. In the DT, specific dual-task instruction was utilized to instruct participants to walk with a concurrent cognitive task (i.e., *“please walk while counting backward by 3’s from [different randomized 2-digit numbers]”*). In the AG, a specific analogy instruction was utilized to instruct participants during walking (i.e., *“imagine you are kicking a ball in front of you”*).

### 2.4. Procedures

A structured questionnaire was first used to collect information on the participants’ characteristics (i.e., age, sex, detailed medical history, detailed fall history, and socioeconomic status). A battery of outcome assessments was subsequently performed to assess the participants’ cognitive and physical abilities at baseline before training (T0) and just after completion of training (T1). All participants took part in three 45 min training sessions per week for four consecutive weeks, resulting in a total of 12 training sessions that were held at local community centers. These training sessions were the only treatment that we provided to the participants. The training sessions were arranged in small groups of five participants and each consisted of (i) 5 min of warm-up and cool down; (ii) 5 min of balance training; (iii) 5 min of body transport training; (iv) 5 min of body transport training with hand manipulation; and (v) 20 min of walking training with various levels of difficulties on a 10 m walkway (i.e., different walking surfaces) under specific instructions as stated by their assigned groups. All training sessions were conducted by registered physiotherapists with experience in geriatric exercise training.

### 2.5. Outcome Assessments

The primary outcome was movement-specific reinvestment propensity, which was assessed by the Chinese version of the Movement-Specific Reinvestment Scale (MSRS-C) [27]. The 10-item scale consisted of two factors: (i) conscious motor processing and (ii) movement self-consciousness. The MSRS has been shown to have high internal consistency and test–retest reliability [7]. The MSRS-C has been validated among Chinese adults through confirmatory factor analysis, which supported the two-factor model previously established in the Western population [27]. A higher score suggests a higher trait movement-specific reinvestment propensity.

The secondary outcomes were functional gait and balance, functional mobility, balance ability, walking ability, and fear of falling. Functional gait and balance were evaluated by POMA [26], a widely recognized clinical assessment tool with good reliability and validity. It can be easily administered to evaluate balance (16 points) and gait (12 points) components simultaneously, contributing to a total score of 28 points [26]. A lower score suggests a higher risk of falling (i.e., ≤18 = high risk; 19–24 = moderate risk; ≥25 = low risk). Functional mobility was assessed by the Timed ‘Up and Go’ Test (TUG) [28]. A duration of over 14 s to complete TUG suggests a high risk of falling for community-dwelling frail older adults [29]. Balance ability was assessed by the Berg Balance Scale (BBS), in which participants were required to perform a total of 14 balancing tasks [30]. A higher BBS score suggests a better balance ability. In terms of walking, single-task and dual-task walking abilities were assessed. Single-task walking ability (in *m*/*s*) was represented by (i) comfortable walking speed for 10 m and (ii) fast walking speed for 10 m [31]. Dual-task walking ability (in *m*/*s*) was represented by (i) comfortable walking speed for 10 m with concurrent verbal secondary task (i.e., auditory Stroop task) [32]; (ii) fast walking speed for 10 m with concurrent verbal secondary task (i.e., auditory Stroop task) [32]; (iii) comfortable walking speed for 10 m with concurrent visual-spatial secondary task (i.e., clock test) [33]; and (iv) fast walking speed for 10 m with concurrent visual-spatial secondary task (i.e., clock test) [33]. Fear of falling was assessed by the falls efficacy scale (FES-13) [34]. A higher score suggests a higher level of efficacy or confidence to participate in regular daily activities without falling [34].

### 2.6. Sampling and Randomization

A sample size of 28 participants per group (total *n* = 84) was calculated to provide sufficient power based on an effect size of 0.31 for the primary outcome of movement-specific reinvestment propensity from a pilot study. After screening 179 potential participants, we found 71 who met the inclusion criteria. We note that we were unable to conduct further recruitment due to the local COVID-19 situation that led to prolonged suspensions of community activities for older adults who were deemed vulnerable to COVID-19.

Recruited participants were randomly assigned to either ST (*n* = 19), DT (*n* = 27), or AG (*n* = 25) using concealed block randomization (i.e., by center) by an independent researcher with an opaque and sealed envelope. An allocation schedule was generated by a computerized random-number generator.

### 2.7. Data Processing

Statistical analysis was performed using SPSS 26.0 (IBM Corp, Armonk, NY, USA). Means (with standard deviation) and numbers (with percentage) were used to describe continuous and categorical outcome variables, respectively. One-way analysis of variance (ANOVA) and chi-square tests were performed to detect between-group differences (ST, DT, AG) in the dependent variables (primary and secondary outcomes) at baseline (T0).

The effects of walking training (ST, DT, and AG) on the primary (movement-specific reinvestment propensity) and secondary (functional gait and balance, functional mobility, balance ability, walking ability, and fear of falling) outcomes across two time points (T0 and T1) were examined using 3 (Group: ST, DT, and AG) × 2 (Time: T0, T1) mixed-model ANOVA with Bonferroni adjusted post hoc tests. The level of significance for all statistical tests was set at *p* < 0.05.

## 3. Results

**Figure 1:**
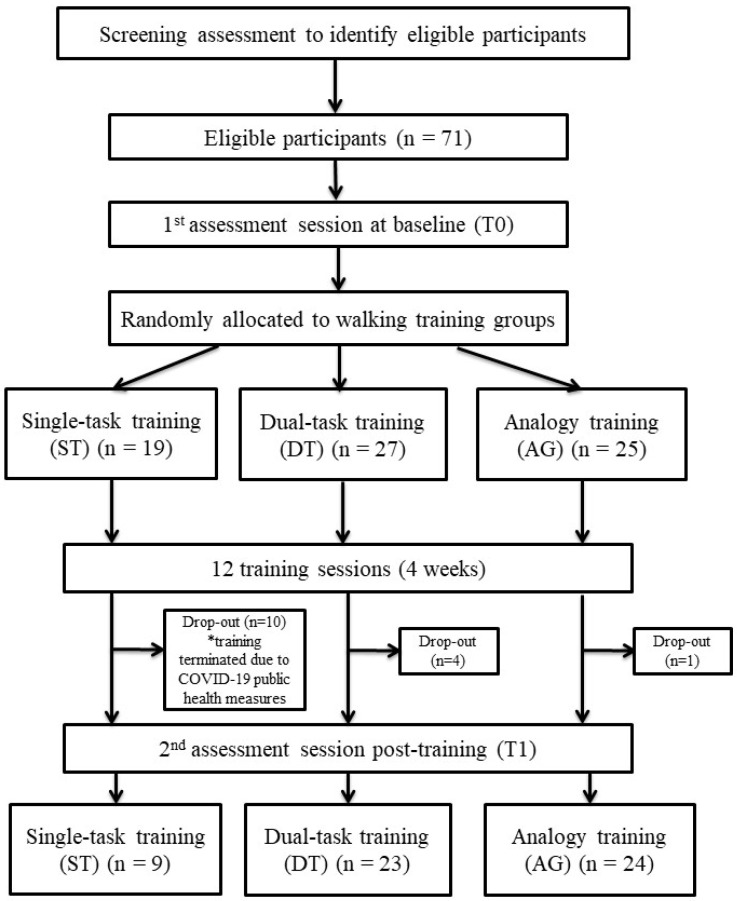
Schematic diagram of the study flow. * Training was terminated due to COVID-19, leading to a high drop-out in ST group.

Figure 1 shows the flow of the study. A total of 56 participants completed the entire training and assessment sessions.

### 3.1. Participants’ Characteristics at Baseline

Table 1 presents the characteristics of the participants at baseline (T0). The mean age of the participants was 82.17 years (SD: 6.63 years). The majority of them were female (*n* = 46, 82.14%) and had a history of falls (*n* = 36, 64.29%). No significant differences among the three groups were observed in any variables at baseline (T0).

### 3.2. Intervention Effects on the Primary Outcome

**Figure 2:**
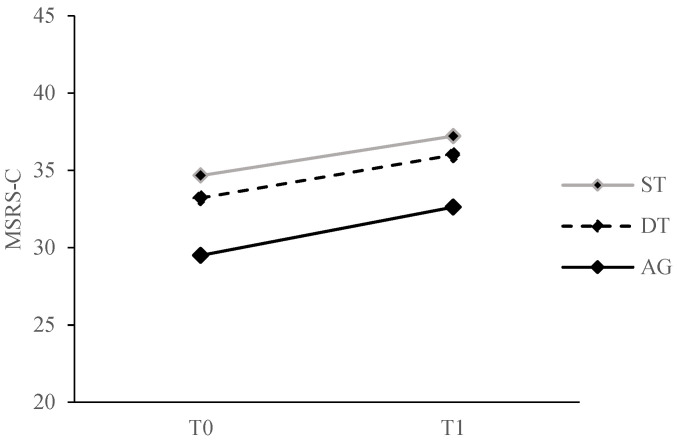
Comparison of movement-specific reinvestment propensity (primary outcome) from T0 to T1 for all training groups. MSRS-C, Chinese version of the Movement Specific Reinvestment Scale; ST, single-task training group; DT, dual-task training group; AG, analogy training group.

There was no significant group × time interaction effect (F[2, 53] = 0.01, *p* = 0.99, ηp^2^ < 0.001) on movement-specific reinvestment propensity. There were no main effects of group (F[2, 53] = 1.01, *p* = 0.37, ηp^2^ = 0.04) or time (F[1, 53] = 1.91, *p* = 0.17, ηp^2^ = 0.04). MSRS-C scores did not differ across groups and time. Figure 2 illustrates the non-significant changes from T0 to T1 in the primary outcome for all groups.

### 3.3. Intervention Effects on the Secondary Outcomes

The results showed no significant group × time interaction effects on the functional gait and balance, functional mobility, and balance ability (POMA: F[2, 53] = 0.94, *p* = 0.40, ηp^2^ = 0.03; BBS: F[2, 53] = 0.46, *p* = 0.63, ηp^2^ = 0.02; TUG: F[2, 53] = 0.05, *p* = 0.95, ηp^2^ = 0.002). There were also no significant main effects of group (POMA: F[2, 53] = 0.30, *p* = 0.75, ηp^2^ = 0.01; BBS: F[2, 53] = 0.44, *p* = 0.65, ηp^2^ = 0.02; TUG: F[2, 53] = 0.10, *p* = 0.91, ηp^2^ = 0.004). There were significant main effects of time, as the POMA, BBS, and TUG scores significantly improved across all groups at T1 compared to T0 (POMA: F[1, 53] = 71.92, *p* < 0.001, ηp^2^ = 0.58; BBS: F[1, 53] = 7.01, *p* = 0.01, ηp^2^ = 0.12; TUG: F[1, 53] = 5.67, *p* = 0.02, ηp^2^ = 0.10).

**Figure 3:**
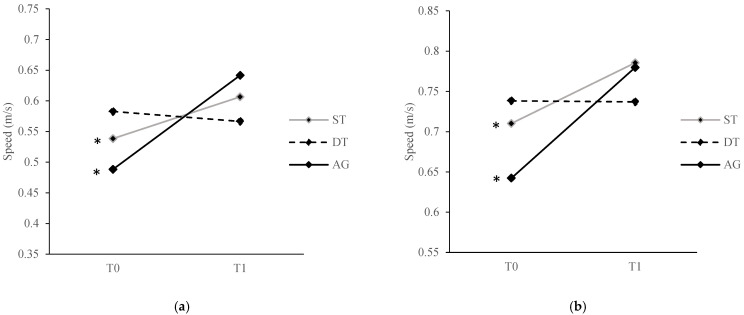
Comparison of single-task walking abilities at (**a**) comfortable speed and (**b**) fast speed from T0 to T1 for all training groups. * denotes *p* value < 0.05 for the difference between T0 and T1. ST, single-task training group; DT, dual-task training group; AG, analogy training group.

**Figure 4:**
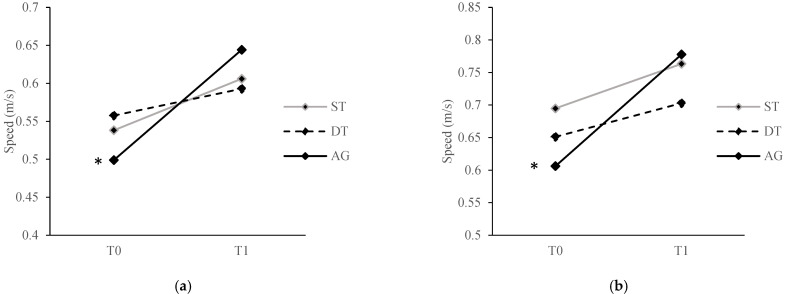

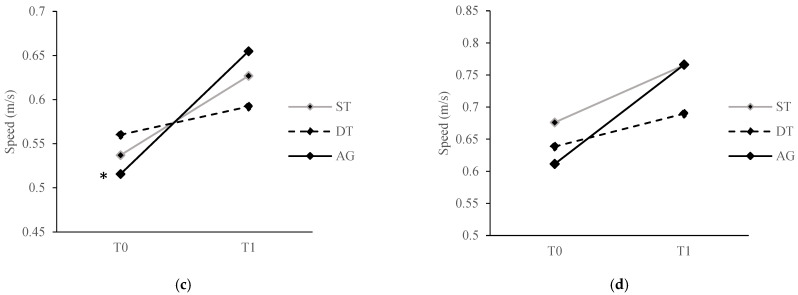
Comparison of dual-task walking abilities at (**a**) a verbal task at a comfortable speed; (**b**) a verbal task at a fast speed; (**c**) a visual-spatial task at a comfortable speed; and (**d**) a visual-spatial task at a fast speed from T0 to T1 for all training groups. * denotes *p* value < 0.05 for the difference between T0 and T1. ST, single-task training group; DT, dual-task training group; AG, analogy training group.

There were significant group × time interaction effects on single-task walking abilities (comfortable speed: F[2, 53] = 8.50, *p* = 0.001, ηp^2^ = 0.24; fast speed: F[2, 53] = 7.06, *p* = 0.002, ηp^2^ = 0.21) and dual-task walking abilities (verbal, comfortable speed: F[2, 53] = 3.86, *p* = 0.03, ηp^2^ = 0.13; verbal, fast speed: F[2, 53] = 5.29, *p* = 0.01, ηp^2^ = 0.17; visual-spatial, comfortable speed: F[2, 53] = 3.36, *p* = 0.04, ηp^2^ = 0.11). Post hoc comparisons revealed that, for single-task walking abilities, ST and AG showed significant improvements in comfortable (ST: t[8] = −3.70, *p* = 0.01; AG: t[23] = −4.44, *p* < 0.001) and fast speed (t[8] = −3.18, *p* = 0.01; AG: t[23] = −4.72, *p* < 0.001) at T1 compared to T0 (Figure 3). For dual-task walking abilities, only AG showed significant improvements in verbal dual-task with comfortable (t[23] = −4.41, *p* < 0.001) and fast speed (t[23] = −5.44, *p* < 0.001) and visual-spatial dual-task with comfortable speed (t[23] = −4.48, *p* < 0.001) at T1 compared to T0 (Figure 4). Post hoc comparisons also revealed that there were no significant between-group differences at T0 and T1 among the three groups for the abovementioned variables (all *p* > 0.05).

There was no significant group × time interaction effect (F[2, 53] = 0.26, *p* = 0.77, ηp^2^ = 0.01), main effect of group (F[2, 53] = 3.93, *p* = 0.05, ηp^2^ = 0.07), or main effect of time (F[1, 53] = 0.49, *p* = 0.61, ηp^2^ = 0.02) on the fear of falling, as the FES−13 scores did not differ across groups and time.

## 4. Discussion

The aim of the current study was to examine the immediate effects of single-task, dual-task, and analogy training on movement-specific reinvestment propensity and fall-related rehabilitation outcomes in older adults with a moderate to high risk of falling. We expected that dual-task and analogy training could mitigate movement-specific reinvestment propensity and yield greater improvements in fall-related rehabilitation outcomes compared to single-task training (active control). Our theoretical assumption was that strategies that promote implicit motor learning could reduce the propensity for movement-specific reinvestment.

Our findings only partially support our hypotheses; we did not find any expected reductions (or increases) in movement-specific reinvestment propensity, as revealed by the MSRS-C scores for all three groups (i.e., ST, DT, and AG) after training. We speculate that the MSRS-C, which quantifies the propensity for trait movement-specific reinvestment, might not be sufficiently sensitive to reflect potentially reduced levels of real-time conscious processing of movements in older adults, particularly in the context of level-ground walking. Although one might argue the possibility that implicit motor learning indeed has minimal effects on the propensity for real-time movement-specific reinvestment, our argument is consistently supported by recent evidence demonstrating the lack of significant association between the MSRS-C score (indicative of trait reinvestment) and EEG T3-Fz coherence (indicative of real-time reinvestment) in community-dwelling older adults during gait tasks [35,36]. Therefore, to better understand the underlying psychomotor mechanism that brings about any changes in rehabilitation outcomes, future studies could consider measuring the real-time conscious processing of movements (e.g., electroencephalography T3-Fz coherence) [36,37,38,39,40]. Such techniques could confirm (or refute) the notion that training methods that promote implicit motor learning could affect real-time movement-specific reinvestment despite trait reinvestment being unaffected.

Despite the lack of significant changes in the propensity for trait movement-specific reinvestment, all three groups showed significant but comparable improvements in functional gait and balance, functional mobility, and balance ability after 12 sessions of walking training. The results suggest that both DT and AG might have similar functional benefits to ST in older adults who are at risk of falling. 

While all three types of training with expertly designed instructions were equally effective at improving functional performance, our findings showed evidence of the advantage of training with an analogy instruction in walking ability among older adults at risk of falling. The AG was found to be superior to the DT and ST in improving walking, as it was the only group that significantly improved both single-task and dual-task walking abilities immediately after training. Analogy is a form of instruction that facilitates skill acquisition by associating it with a fundamentally similar concept [41,42,43]. Analogy learning is considered an implicit motor learning strategy, with minimal demand on attentional resources. When less demand is placed on cognitive resources during skill acquisition, more resources would then be available for consolidating the structure of the motor skill [21]. More robust motor performance has been observed in previous studies when analogy instruction was provided in cognitively demanding situations, such as stressful conditions or dual-task contexts [44,45,46,47]. Our findings are in line with previous literature [48,49], suggesting that individuals who received analogy training have largely acquired the skill in an implicit manner and, more importantly, are able to demonstrate the transferability of the concept of analogy learning to other contexts (e.g., different dual-task conditions). In addition to aiding the understanding of movement instructions for older adults whose cognitive function declines with age, these advantages of movement analogies might be attributable to the mental representations developed in long-term memory [50], the simplicity of recalling analogies from memory [51], and lower demands related to information processing (i.e., reduced dependence on working memory) [12,50].

When considering the outcomes of walking ability through other training groups, ST only showed significant improvements in single-task walking speeds, while DT did not show any improvements in all measures of walking ability. While it was reasonable to expect improvements in (single-task) walking speeds after a 4-week group-based training targeting solely walking (ST), our findings might hint at the notion that walking training under single-task contexts might not be able to generalize to locomotor control during dual-task conditions [52]. For dual-task training, it remains unclear why DT did not improve single-task or dual-task walking abilities, but we postulate that older adults might only be able to reflect training-specific improvements in dual-task performance since dual-task learning is likely task-specific [53]. In our case, the DT trained the cognitive task of counting backward by threes, while the assessments of dual-task walking ability included an auditory Stroop task and a clock test. Previous findings appear to indicate the importance of training specificity. The transfer of training benefits from a specific type of dual-task training to another has been consistently lacking [54,55], including in our current findings. However, given that dual-task walking abilities have been shown to be associated with fall risks [56], the lack of such transferability might prove to be critical to functional independence for older individuals, as dual-task walking is commonplace in daily living, where different secondary tasks likely occur during locomotion (e.g., walking while talking). As such, when viewing our overall evidence that only AG resulted in significant improvements in single-task and dual-task walking abilities, we suggest that training with expertly designed analogy instructions is a relatively more compelling choice for the gait re-education or rehabilitation of at-risk older adults.

Several limitations should be taken into account when interpreting the results. First, the study was severely impacted by public health measures in response to COVID-19 in Hong Kong. Hence, we were not able to meet our target sample size, and there was a high drop-out number for the ST group, leading to an imbalance in sample sizes among the three groups (i.e., the sample size for ST was unexpectedly small). Unequal sample sizes might potentially affect statistical power and the risk of type I error. Second, one cannot neglect the possibility that older adults were using previous locomotor experience and/or adaptive learning strategies rather than solely relying on the instructions provided by us during training. Another limitation was the use of gait speed alone to quantify the performance of walking abilities under single-task and dual-task conditions. Gait speed has been shown to be a good predictor for falls [57,58], physical performance [59,60], and mortality [61]. However, further investigations with alternative behavioral outcomes (e.g., spatial and/or temporal parameters of gait, stability measures, etc.) may provide other meaningful aspects of walking abilities. Fourth, we acknowledge that a greater number of training sessions might be better for the improvement of any outcomes. Nevertheless, the current duration and frequency of training sessions were referenced from the usual clinical practice of physiotherapy for geriatric rehabilitation in Hong Kong. Finally, we planned to implement a retention test at six months post-training. However, due to the continuing impact of the pandemic in Hong Kong, we had to prematurely terminate the trial. Further research is recommended to examine the long-term retention of improvements in walking abilities.

To the best of our knowledge, this is the first RCT that compared the immediate effects of single-task, dual-task, and analogy training on movement-specific reinvestment propensity and fall-related rehabilitation outcomes in older adults. Our findings, while tentative, have potential implications for the design and development of psychomotor interventions that aim to enhance the wellbeing of older adults through fall prevention. Implementing the concept of analogy learning could be a feasible and effective intervention in geriatric rehabilitation settings. Physiotherapists could consider using analogies as a way of modifying instructions for older adults at risk of falling. Further research is recommended to strengthen the evidence and support implementation in practice.

## 5. Conclusions

The present study revealed that walking training through single-task, dual-task, and analogy strategies significantly improved the functional outcomes (i.e., functional gait and balance, functional mobility, and balance ability) immediately after training. There were no significant effects on movement-specific reinvestment propensity by any training strategy. Using analogy training appeared to yield significant improvements in both single-task and dual-task walking abilities. We provide evidence for the notion that walking training with analogy instruction is a promising approach that has the potential to improve functional walking outcomes in older adults at risk of falling. For geriatric rehabilitation, our findings might be considered for service provision, where analogy instructions during gait training could potentially contribute to the prevention of falls and the promotion of wellbeing.

## Figures and Tables

**Table 1:** Participants’ characteristics at baseline (T0).

Variables	Mean (SD)				*p* Value
	Total (*n* = 56)	ST (*n* = 9)	DT (*n* = 23)	AG (*n* = 24)	
Age	82.17 (6.63)	80.11 (7.08)	81.96 (6.51)	82.38 (6.36)	0.67
Sex, male, *n* (%)	10 (17.86)	2 (22.22)	4 (17.39)	4 (16.67)	0.93
With a history of falls, *n* (%)	36 (64.29)	6 (66.67)	18 (78.26)	12 (50.00)	0.13
MSRS-C	32.07 (12.78)	34.67 (12.43)	33.22 (11.19)	29.50 (14.40)	0.48
POMA	20.72 (2.71)	21.33 (1.80)	20.43 (2.71)	20.83 (3.07)	0.70
BBS	43.12 (6.40)	44.11 (4.43)	42.48 (6.16)	43.79 (7.14)	0.72
TUG (seconds)	21.95 (8.30)	21.55 (7.16)	22.45 (7.04)	21.64 (10.21)	0.94
FES-13	100.90 (21.67)	104.89 (15.17)	99.00 (20.98)	101.63 (24.50)	0.78
*Single-task walking ability*					
Comfortable speed (m/s)	0.54 (0.20)	0.54 (0.17)	0.58 (0.25)	0.49 (0.16)	0.29
Fast speed (m/s)	0.69 (0.25)	0.71 (0.22)	0.74 (0.32)	0.64 (0.18)	0.42
*Dual-task walking ability*					
Verbal, comfortable speed (m/s)	0.53 (0.20)	0.54 (0.20)	0.56 (0.24)	0.50 (0.16)	0.60
Verbal, fast speed (m/s)	0.64 (0.24)	0.69 (0.25)	0.65 (0.30)	0.61 (0.19)	0.63
Visual-spatial, comfortable speed (m/s)	0.54 (0.22)	0.54 (0.19)	0.56 (0.28)	0.52 (0.17)	0.79
Visual-spatial, fast speed (m/s)	0.64 (0.24)	0.68 (0.21)	0.64 (0.29)	0.61 (0.18)	0.78

Note. ST, single-task training group; DT, dual-task training group; AG, analogy training group; MSRS-C, Chinese version of the Movement Specific Reinvestment Scale; POMA, Tinetti Performance Oriented Mobility Assessment; BBS, Berg Balance Scale; TUG, Timed ‘Up & Go’ Test; FES-13, Falls Efficacy Scale; SD, standard deviation.

## Data Availability

The data that support the findings of this study are available from the corresponding author upon reasonable request.
